# The relevance of Spiritual Leadership to public health: values, meaning and purpose

**DOI:** 10.3389/fpubh.2025.1632959

**Published:** 2026-01-28

**Authors:** Louis W. Fry, Barbara X. Wei, Fawn Phelps, Richard B. Siegrist, William Bean, Theodore J. Witherell, Howard K. Koh

**Affiliations:** 1 Texas A&M University, Central Texas, Killeen, TX, United States; 2 Health Policy and Management, Harvard T.H. Chan School of Public Health, Boston, MA, United States

**Keywords:** Public Health Leadership, Spiritual Leadership, public health competencies, leadership development, public health training and education

## Abstract

Public health currently faces many enormous challenges that highlight the urgent need for more effective leadership models. This paper explores Spiritual Leadership in the Workplace as a potential approach to informing and animating the field of Public Health Leadership. Developed and applied over the past two decades, Spiritual Leadership is defined as the “values, attitudes, and behaviors necessary both to motivate and inspire workers and to enhance key individual and organizational goals through a vision of service and a culture based on altruism.” While for some it can relate to, and build upon, religious practices, Spiritual Leadership more broadly relates to leadership based on personal values and actions emerging from a sense of mission, purpose, and connection to something bigger than oneself. This paper first describes the origin and evolution of Spiritual Leadership and its necessary and organizational development competencies. Then, we conduct a literature review about Public Health Leadership competencies and find that many of them map directly onto the Spiritual Leadership model, thereby opening an opportunity for integration. We also acknowledge both opportunities and challenges for Spiritual Leadership implementation in secular and culturally diverse settings while noting substantial anecdotal indications that many public health leaders already enact Spiritual Leadership-consistent practices. We conclude with a discussion on implications for Public Health Leadership education, workforce development, and research. Spiritual Leadership can serve as a unifying, practice-oriented leadership perspective that can help strengthen purpose, belonging, and resilience in public health organizations.

## 1 Introduction

In recent years, the longstanding challenges of public health leadership have been on full global display. The field, long recognized as highly complex, is lately even more riddled with divisive challenges that have spilled over into public view. Controversies regularly arise about how best to distribute finite resources to address seemingly infinite health needs (1). Volatile conflicts can divide the involved parties that regularly include powerful vested interests. A coordinated response usually, if not inevitably, falls outside the control of a single authority (2). During the pandemic, these kinds of challenges, debated daily, left public health leaders facing resistance and threats which accelerated burnout, and complicated worker retention and recruitment (3). Now, major uncertainty and disruption stemming from substantial public health changes being made nationally further highlight the pressing need for more effective public health leaders.

New leadership paradigms can help animate the future of Public Health Leadership especially as the post-pandemic world demands shifting from acute crises to sustained crises (4). While much has been written about leadership in professions such as business, the military, and sports, Public Health Leadership still remains a relatively new field of scholarly inquiry. Understanding, and potentially applying, established models and frameworks developed for other societal sectors can stimulate education and research innovation. In a recent article, two of us (HK, FP) proposed that exploring the spiritual foundations of Public Health Leadership could inform future education, training, and research. “Spirituality” has been defined in one international consensus conference as “a dynamic and intrinsic aspect of humanity through which persons seek ultimate meaning, purpose, and transcendence, and experience relationship to self, family, others, community, society, nature, and the significant or sacred.” Indeed, public health is regularly viewed by its practitioners as a “calling,” imbued with a sense of mission, service, and connection to something bigger than themselves (5).

However, spirituality has not yet been widely recognized in an explicit way as an essential aspect to leadership in general and in public health in particular. For example, the three most widely adopted, leadership-focused textbooks—Northouse’s *Leadership: Theory and Practice* (2024) (6). Daft’s *The Leadership Experience* (7), and Yukl & Gardner’s *Leadership in Organizations* (2020)—cite the importance of values-based models with an emphasis on moral principles and ethical conduct (7–8) such that leadership is authentic (9), transformational (10), and/or ethical (11). In addition, servant leadership, which has deep roots in Christian leadership traditions (12–14), emphasizes service, humility, and follower wellbeing. But all of these important models may not fully qualify as a comprehensive model of workplace spirituality (15). Even the critical and invaluable model of servant leadership could benefit from more attention to an operational set of core values to guide practice; a clearer approach to aligning values, attitudes, and behaviors across individuals, teams, and organizations; clarification of how to balance follower needs with organizational goals; and greater attention to outcomes for both leaders and followers.

Such themes are addressed through Spiritual Leadership, a model developed within business and organizational management over the past 25 years (15). Spiritual Leadership holds that spirituality is necessary for religion, but religion is not necessary for spirituality. At its core, spirituality reflects the human quest for transcendence through the energy drawn from one’s values and beliefs, producing a sense of interconnection and harmony with others (16, 17). While these themes are often expressed within religious traditions, Spiritual Leadership frames them in broader and more inclusive terms. As such, it provides a spiritual approach to leadership—grounded in vision, values, and the search for ultimate meaning and purpose—that can resonate with individuals from many faiths—or with no formal religious affiliation at all (18).

Spiritual Leadership uniquely theorizes a pathway through hope and faith in a vision of serving key stakeholders within a culture anchored in altruistic values. This process addresses fundamental human needs for spiritual wellbeing by cultivating calling (meaning and purpose) and membership (belonging and community), which together support both individual flourishing and organizational effectiveness. In doing so, Spiritual Leadership incorporates and extends servant leadership, for example, by explicitly operationalizing values such as integrity, compassion, forgiveness, humility, and courage; by making vision and value congruence a central leadership objective across organizational levels; and by specifying calling and membership as intrinsic motivational mechanisms. In this way, Spiritual Leadership reconciles the care for followers with the pursuit of organizational performance, offering a more fully elaborated framework for leadership in the modern workplace.

Spiritual Leadership also has the potential for addressing pressing public health leadership challenges: burnout remains a major post-pandemic challenge for the workforce and positive working conditions can lead to better mental health (19); the *National Academy of Medicine’s National Plan for Health Workforce Wellbeing* calls for restoring meaning, joy, and fulfillment in work (20); and the *WHO’s Framework for Action on Interprofessional Education & Collaborative Practice* emphasizes shared purpose and relational trust as prerequisites for team-based care (21). By making spiritual wellbeing (meaning and belonging) the explicit driver of culture and performance, Spiritual Leadership offers a competency-based framework to address these challenges.

In this article, we review the key concepts and evolution of Spiritual Leadership, a model uniquely and explicitly focused on spirituality, mission, vision and values, and explore its relevance to public health (18). Originally proposed and developed over the past two decades by one of the co-authors (LF), the Spiritual Leadership framework contends that spirituality, while inherently personal, can serve as an explicit foundation for leadership in the workplace. We begin by describing the model and its necessary elements and competencies (i.e., key qualities contributing to organizational success). We also note the model’s evolution in the fields of organizational development (OD) and business and highlight the major published studies and meta-analyses linking it to relevant individual, organizational, and stakeholder outcomes (22–25). We then discuss how the necessary and organizational development competencies can inform Public Health Leadership, noting not only the opportunities but also acknowledging challenges and nuances involved. Finally, we consider possible ways of applying the Spiritual Leadership model to Public Health Leadership research, education, and practice.

## 2 Spiritual Leadership

**Figure 1:**
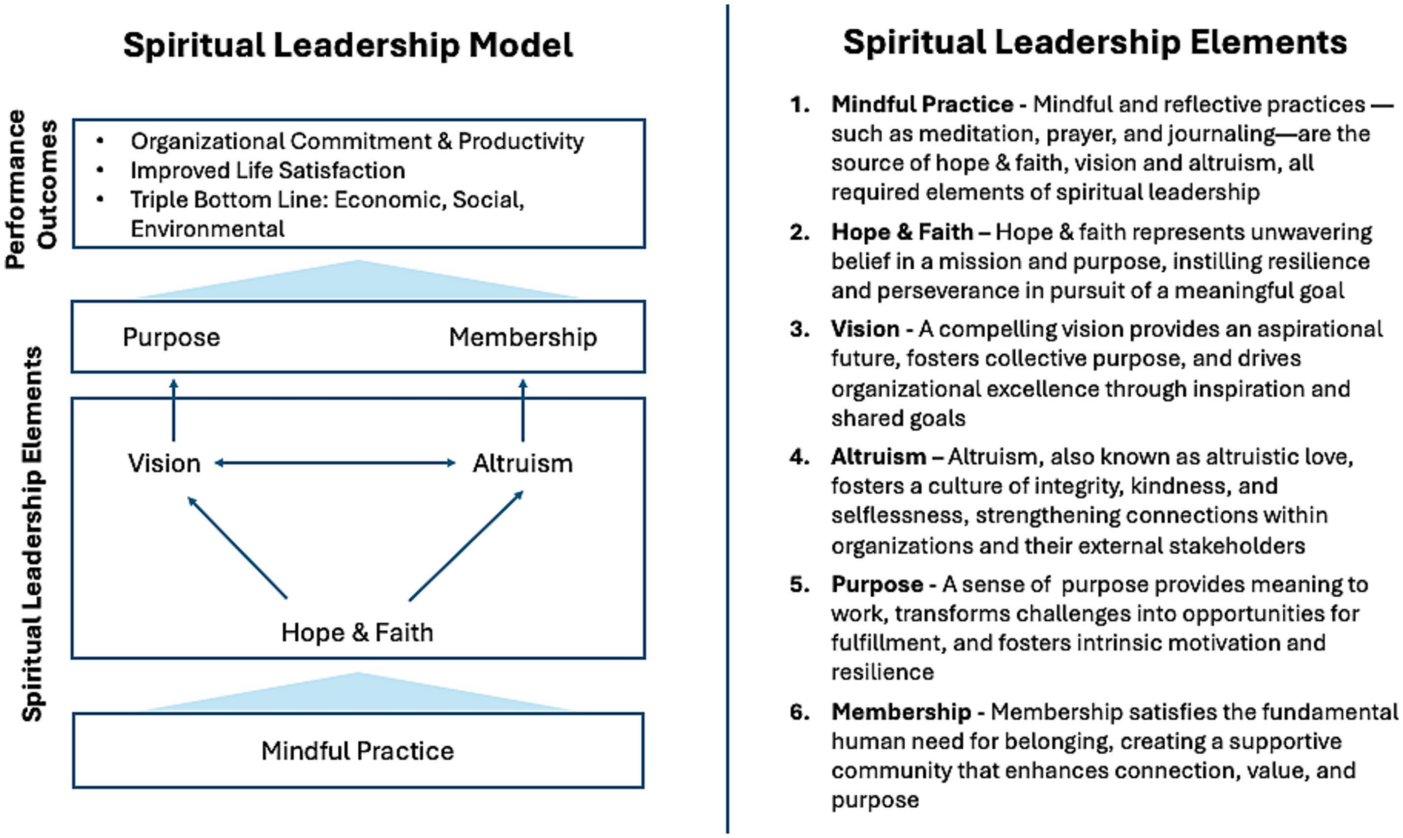
Spiritual Leadership model.

In 2013, Fry and Nisiewicz wrote a foundational book describing six interrelated elements, as well as the necessary and organizational development competencies, required of leaders to align the values of their organizations with the individuals who work in them. They argued that doing so can further organizational performance goals or outcomes, such as employee wellbeing and life satisfaction, productivity, and social and environmental responsibility (22, 23, 26). As Figure 1 describes, the first of these elements, *mindful practice*, enables self-awareness and the ability of leaders to draw strength from a power greater than themselves. That element also facilitates *hope* and *faith* in a *vision* shared throughout an organization, which, in turn, creates a sense of *purpose* fostering intrinsic motivation. A culture of *altruism* (aka “altruistic love”) creates a sense of *membership* in a community that supports this purpose. By encouraging individuals to first understand themselves and by facilitating shared reflection among all parties, the Spiritual Leadership model shapes collaborative, discerning, and creative choices in times of decision. It can also reveal assumptions limiting the ability to hear and appreciate different points of view, essential for building trust.

## 3 Evolution of the Spiritual Leadership model

Over the past two decades, the Spiritual Leadership model has evolved into a robust framework supported by a growing body of empirical evidence (Table 1).^1^ The pioneering work of Mitroff and Denton first allowed the concept of Spiritual Leadership to gain recognition as a legitimate area for academic research (27). They theorized that recognizing these spiritual needs in the workplace positively influences employee health and psychological wellbeing (28). Subsequent work established and extended this concept. In a 2005 special issue of the *Leadership Quarterly* dedicated entirely to Spiritual Leadership, Fry expressed his aim to advance Spiritual Leadership toward “paradigmatic status” as a comprehensive framework for organizational transformation (29).

**Table 1:** Timeline of the development of Spiritual Leadership model.

Year	Event/Development	Details
1999	Mitroff & Denton’s pioneering work (27)	This foundational work in the field of workplace spirituality explores how spiritual values intersect with corporate culture and performance
2005	Special issue of The Leadership Quarterly on Spiritual Leadership (33)	Fry and colleagues aim to establish Spiritual Leadership as a paradigm for organizational transformation
2005–2012	Further Spiritual Leadership Model development (37, 68–74)	Explores the relationship between Spiritual Leadership and ethical and spiritual well-being. Adds “inner life” as the source of Spiritual Leadership, which is then expanded to Levels of Being; Articles address egotism and workaholism, character development, and Spiritual Leadership impact on performance outcomes
2013	Explored Spiritual Leadership and maximizing the triple bottom line (26)	Reviews the emerging fields of workplace spirituality and Spiritual Leadership to show how business models address employee well-being, social and environmental responsibility while maintaining metrics of performance excellence
2017, 2023	Incorporation of non-Western spiritual traditions (75, 76)	Transposes Spiritual Leadership theory into an Islamic leadership model that better aligns with the values of Islamic organizations and Muslim employees Integrates the Buddhist concept of non-self into Spiritual Leadership theory and reveals how egoistic attachments can lead to pseudo-Spiritual Leadership
2017, 2022	Expands on inner life as the source of Spiritual Leadership (46, 77)	Explores how a Global Mindset develops, emphasizing that self- and other awareness arise from progressing through levels of being in a Spiritual Leadership framework Presents a framework linking spiritual, moral, and leader development through shared markers and practices to support Spiritual Leadership growth
2021	Fry & Egel’s Global Leadership for Sustainability (GLfS) model (78)	Proposes the Global Leadership for Sustainability (GLfS) model with Spiritual Leadership as the foundation, to promote self-transcendence, a global mindset, and ethical principles that prioritize the triple bottom line over purely economic outcomes
2024	Expands GLfS to leading transformative multi-sector partnerships (79)	Identifies leadership competencies for transformative multi-sector partnerships (MSPs) and shows how the Global Leadership for Sustainability (GLfS) model incorporates them to support sustainable development and the triple bottom line

A period of initial testing and validation of the model followed. In 2013, scholars of business development began publishing studies about ways to incorporate personal spiritual journeys, including spiritual concepts from outside the Western tradition from the Dalai Lama and Buddhist philosophy, for example, into organizational culture. Mindfulness has taken center stage as the source of Spiritual Leadership. Thich Nhat Hanh, known as the Father of Mindfulness, wrote that “you cultivate the energy of mindfulness with mindful breathing and mindful walking, and with that energy, you can recognize and tenderly embrace your worry, fear and anger” (30). The most recent work has focused on ways Spiritual Leadership might enhance business leadership on the global stage, with particular attention to sustainable development. These principles encourage business leaders to extend considerations beyond immediate stakeholders and financial outcomes to broader community and environmental wellbeing highlighting the “triple bottom line” (social, environmental, financial). Table 1 offers a timeline of the development of the Spiritual Leadership model.

### 3.1 Significant Spiritual Leadership empirical studies

**Figure 2:**
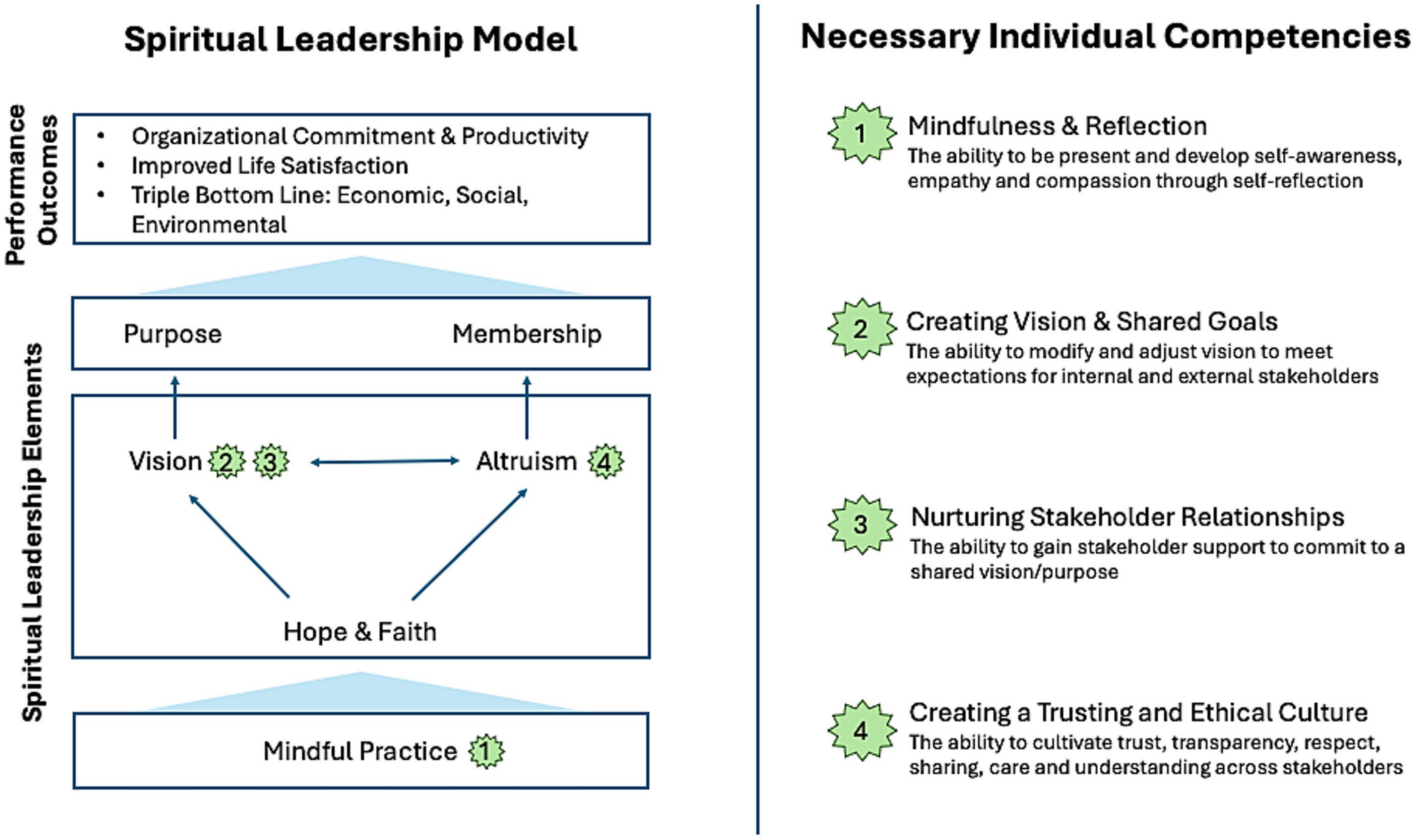
Spiritual Leadership competencies.

The Spiritual Leadership model has steadily evolved over two decades, supported by growing empirical evidence (Table 2). Numerous studies using structural-equation modeling have confirmed how its six core elements (see Figure 2) influence both organizational and individual outcomes (31, 32). This method reveals both direct causal pathways and complex interrelationships. A highly reliable and valid Spiritual Leadership survey, now widely used in empirical research, has been applied over 20 years across diverse organizations (See section 7.1 and Appendix 1) (23).

**Table 2:** Timeline of significant empirical studies confirming the Spiritual Leadership model.

Year	Event/Study	Findings
2005	Ft. Hood helicopter squadron longitudinal study (31)	Used structural-equation modeling to support reliability and validity of Spiritual Leadership measures and establish baseline for future organizational development interventions
2013	Spiritual Leadership tested in South Korean for-profit companies (34)	Showed that in South Korean firms, inner life boosts key Spiritual Leadership factors, with membership having double the impact of calling on productivity and life satisfaction
2017	Spiritual Leadership Study on Baldridge Award-winning leaders (35)	Tested the Spiritual Leadership model with a sample of U.S. Baldrige award recipients and revealed strong links between Spiritual Leadership and outcomes essential to performance excellence
2018	Healthcare study on burnout and commitment (32)	Found Spiritual Leadership improves commitment, productivity, and life satisfaction, while reducing burnout among medical lab personnel
2019	Study on Chinese healthcare organization (36)	Identified that relational energy mediates the link between Spiritual Leadership and job performance, with leader integrity strengthening, and energy differentiation, weakening this effect
2020	Oh & Wang survey and meta-analysis (23)	Reviewed 59 studies and analyzed broad factors and outcomes related to Spiritual Leadership; Confirmed high validity and reliability.
2024	Literature review of 286 publications (38)	Analyzed 286 Scopus-indexed articles on Spiritual Leadership using citation, co-citation, and cluster analysis to identify key themes, influential works, and five major research streams
2024	Vedula & Agrawal review of 337 published papers (1980–2021) (80)	Analyzed 337 papers from 1980 to 2021, using bibliometric and content analysis, to map key themes in Spiritual Leadership, highlight major contributors, and suggest future research

Researchers have studied Spiritual Leadership in diverse workplace contexts. A foundational 2005 longitudinal study at Ft. Hood, Texas validated Spiritual Leadership’s measures and informed later interventions (31). Many studies link Spiritual Leadership to enhanced organizational commitment and productivity through increased engagement, belonging, and life satisfaction (26, 33). The revised “inner life” model (2009) further supports these links. These studies span different geographies and populations, including a 2013 South Korean study (34); a 2017 seminal study on winners of the Baldridge Award, the highest level of national recognition that a US organization can receive for performance excellence given by the National Institutes of Science and Technology (NIST) (35); and a 2019 study of hotel staff (36).

Extensive reviews and meta-analyses summarize the current status of the model’s reliability, validity, and broad workplace impact (23, 26, 37, 38). Oh and Wang reviewed 59 studies showing Spiritual Leadership outcomes are shaped by internal and external factors (23). A 2024 Scopus-based review of 286 articles detailed the model’s antecedents, moderators, and mediators (38) and a separate statistical analysis of 373 books, articles and publications (1980–2021) affirmed Spiritual Leadership’s role in fostering sustainable workplaces (34). The Oxford Dictionary now includes Spiritual Leadership as a recognized leadership concept (39).

## 4 Necessary Spiritual Leadership competencies

Necessary Spiritual Leadership competencies (Figure 2) represent the knowledge, skills, and personal attributes essential for effective outcomes (26, 40–44). With the overall goal of enhancing performance, adaptability, and wellbeing, Spiritual Leadership competencies inform a process of organizational change to improve overall effectiveness (42). Ultimately, it aligns people, processes, and resources with the organization’s vision and strategic goals (42).

### 4.1 Mindfulness & reflection competency

“Mindful” or “reflective” practices can nourish leaders trying to reach the “elevated” state of consciousness, or self-awareness needed to cultivate and practice Spiritual Leadership. In the Spiritual Leadership model, such practices involve a wide array of options that can be personalized, ranging from simple to complex. They include reflection in silence, solitude in nature, meditation, journaling, readings, creating art or music, prayer, Tai Chi, yoga, chanting, visualizations and affirmations, pilgrimage, service or even fasting. The chosen practice helps the leader focus on the current situation (“become more present in the moment”) rather than dwell on the past or the future. Thich Nhat Hanh, the father of mindfulness, highlights the use of breath to recognize affliction and stay in the present moment (45). The competency also helps foster a work environment that supports mindful & reflective practices in others (26). These practices can also help leaders relinquish the belief that the ego and intellect can resolve issues created by the pursuit of personal power, prestige, and wealth (46–48). Shifting values and priorities away from the trappings of personal success and economic gain can cultivate active observation of present thoughts, emotions, and bodily sensations. This heightened self-awareness, or “informed mindfulness” can foster greater compassion and empathy, self-regulation, resistance to anger and other negative emotions, and educated decision-making (46).

### 4.2 Creating vision & shared goals and nurturing stakeholder relationships competencies

Co-creating a compelling and inspirational vision can foster collective purpose that drives organizational success. For that reason, the competencies of creating vision & shared goals and nurturing stakeholder relationships are inextricably linked. Cultivating relationships with stakeholders allows leaders to better understand diverse perspectives, facilitate more informed decision-making and strategy development (49–51), and ensure that the vision is achievable, not just aspirational (52).

To strike a balance between ambition and practicality, engaging and understanding stakeholders can align the vision internally within an organization and externally. Neglecting either the organization’s or external stakeholders’ perspectives can invite resistance and conflict. Hence, a formal vision and stakeholder analysis can simultaneously align the organizational vision while nurturing relationships with all parties, uncover new opportunities, foster buy-in and lead to enhanced engagement with partners that can lead to innovation (53, 54).

### 4.3 Creating a trusting and ethical culture competency

Cultivating altruistic values—such as integrity, patience, kindness, forgiveness, acceptance, gratitude, humility, courage, loyalty, and compassion—help create a trusting and ethical culture. The literature consistently highlights three common qualities in leaders with this competency:

- Promoting transparency and respect for diverse voices
- Sharing information and resources and demonstrating integrity and follow-through
- Developing shared understanding of problems, and effective working relationships that reinforce collective values (26)

Such practices understand and leverage the fundamental need of membership of both leaders and followers.

### 4.4 Organizational development competencies: building teams, managing change, collaborative decision making and managing conflict

Organizational development, a planned, evidence-based discipline that enhances an organization’s effectiveness, adaptability, and member wellbeing regularly emphasizes that four outcomes applicable to all effective organizations are building teams, managing change, promoting collaborative decision making and managing conflict (26). Collaboration within and across groups can facilitate smooth transitions during periods of change, promote inclusive approaches to input and shared decision-making, and foster constructive methods for addressing disagreements and workplace tensions. By advancing these relational and operational capabilities, organizational development seeks to create a more resilient, aligned, and high-performing organization (40–44, 55, 56).

## 5 Adapting and translating the spiritual leadership model to public health

More attention to spiritual grounding can be valuable in navigating the complexities and challenges of Public Health Leadership (5). An approach emphasizing the interconnectedness of values, meaning, purpose, mindfulness, and leadership can cultivate more resilient, compassionate, and effective public health leaders. Spiritual Leadership in the public health context has the potential to enhance staff engagement and satisfaction by fostering spiritual growth and wellbeing for all those in the field trying to mobilize people to make change. For direct healthcare professionals, prompting them to understand and acknowledge these themes can be useful for both them and also their patients, especially in times of serious illness (57).

Adapting the Spiritual Leadership model to the public health workplace offers a way to deepen this conversation, while introducing intriguing, and often nuanced and challenging, opportunities. Baber and Baber (70) have emphasized that creating a spiritually supportive work environment within healthcare organizations should be viewed not just as a personal attribute, but also as a crucial component of organizational health and effectiveness.

Despite this clear potential, successfully applying Fry’s model to public health education, research, and practice will require certain adaptations. Starting by modifying some of the wording can allow stronger resonance with a larger public health audience. More research can explore which elements of the Spiritual Leadership model most resonate with public health leaders. Indeed, the term “spiritual leadership” itself may engender hesitancy in some public health practitioners due to negative personal experiences with religion or wariness about how religious differences have contributed to conflict and wars through history. Educators may also be concerned about the longstanding caveats associated with the Establishment Clause of the Constitution that serves as the basis of separation of church and state.

Pre-empting these reactions could start by emphasizing that Fry’s model addresses a broad definition of spirituality that does not necessarily equate to religion. Currently, over 90% of the US public believes in the existence of a soul, something spiritual beyond the natural world, while ~50% consider themselves religious (58). The broader Public Health Leadership community can more readily engage in discussions about their values, core beliefs and sense of mission and calling which has broad appeal to anyone feeling a connection to something greater than themselves. Doing so helps all aspiring public health leaders crystallize what gives them ultimate meaning and purpose in their work.

Equally important will be adapting some of the terms used in the Spiritual Leadership model to apply more directly to public health. The element of “mindful practice,” for example, might be translated more concretely as “developing self-awareness.” The term “membership” in Fry’s model, which refers to a sense of belonging, relates to “community.” Fry’s original term “altruistic love,” potentially difficult to incorporate into data-dominated public health practice, might be adapted simply to “altruism”—a concept reflecting a person’s concern for both self and others. Such a concept fits comfortably into the practice of Public Health Leadership, which inherently involves building unity among those separated by health status, income, structural barriers to health, and other disparities.

Still other terms like “hope & faith” might be more effectively described as an “unwavering belief in a mission and purpose, instilling resilience and perseverance in pursuit of a meaningful goal.” As opposed to “optimism” which implies that the external odds are likely to change for the better, “hope” implies an internal belief in a positive outcome regardless of odds. Meanwhile, “faith” implies a sense of perseverance and commitment to the journey, however difficult. As Yale University Chaplain Reverend William Sloane Coffin, a civil rights and antiwar campaigner, once said, “Hope puts you on the road, but faith keeps you there” (5). Some combination of hope, faith and optimism can empower public health leaders to more fully live the mission that fulfills the larger purpose of improving population health and wellbeing.

Other elements of the Spiritual Leadership model may resonate more readily if focused more explicitly on aspects of public health. The element of purpose, for example, can be relevant in addressing some basic Public Health Leadership questions such as “What are you good at?,” “What does the world need from you?,” and even “What brings you joy?” (59). While public health leaders may not necessarily experience “happiness” as they struggle to execute the hard things required of them, they can nevertheless feel joy as a “sense of deep delight in being called to something before you” (59). In a recent documentary, Archbishop Desmund Tutu captures the power of such joy in describing the ability of “Doctors Without Borders to go into hugely dangerous situations with an outpouring of love, compassion, and caring for people they do not know. We are wired to be caring for each other” (60).

## 6 Implications for education

Public Health Leadership programs regularly focus on external skills, like strategic thinking and time management, while sometimes overlooking the critical inner motivation and purpose required to execute such skills effectively. In public health, where interventions must always adapt to shifting evidence, funding and frontline feedback, leaders can create spaces that welcome innovation and even risk failure. The Spiritual Leadership framework can facilitate connecting passion with purpose and build upon the foundation of calling to sustain leaders. With greater clarity about purpose, leaders can leverage emotional resilience in the face of complexity, uncertainty, and potential burnout.

Most aspiring public health practitioners inevitably wrestle with identifying their precise role in upholding the broad lofty, and sometimes overwhelming, mission of public health. Their very personal decisions about when and how to respond to a call to lead are essentially spiritual acts. Teaching students to explore their personal “why” can help them understand their motivations and connect more deeply with others and navigate the challenges of decision making (61). While discussions around faith and spirituality in classrooms can be sensitive, they can also highlight shared values like service and empathy that resonate across belief systems. Gupta has written in his book *Bridges Across Humanity* that in fact, all the major religions share 54 common themes including compassion, service, unity and emphasis on caring for the vulnerable—all these themes relate to Public Health Leadership (18). Developing the necessary Spiritual Leadership competencies can reduce reactivity and set the stage for emotional regulation, creative problem-solving, and reflective decision-making, the currency of modern public health leadership.

The mindfulness and reflective practices noted earlier in Section 4.1 not only draw from myriad wisdom traditions but also align with established leadership frameworks such as emotional intelligence, authentic leadership, adaptive leadership, and key concepts like psychological safety and transformative learning. Among many foundational ideas grounding this approach is Warren Bennis’ classic work on “crucibles of leadership,” which shows how proven leaders have often endured unanticipated, intense, traumatic experiences that forced re-examination of basic values to emerge more committed to mission. Such leaders who endure such experiences can find renewed, even heightened, meaning in their work, while understanding, as Coffin has said “giant obstacles are brilliant opportunities, brilliantly disguised as giant obstacles!” (62). Another framework, Ronald Heifetz’s adaptive leadership model, intertwines the values of Spiritual Leadership and Public Health Leadership in using self-awareness to mobilize people to tackle complex and ever-evolving challenges, often through loss and discomfort, that cannot be solved through technical expertise alone (63). Similarly, adrienne maree brown’s [sic] *Emergent Strategy*, rooted in decades of community organizing, provides practical guidance for cultivating trusting and ethical cultures, a core spiritual leadership competency especially relevant for public health leaders seeking to align interventions across self, family, and community, and systems (64).

## 7 Implications for research

Two of us (BW, LF) conducted a literature search on Public Health Leadership using Scopus, Web of Science and the EBSCO host platforms, covering publications from database inception through our last search on August 23, 2025. Our search used controlled vocabulary and free-text terms related to public health and leadership competencies/management/governance, including the following terms: public health leadership, public health leadership competencies, spirituality, transcendence, interconnectedness, mindfulness, purpose, belonging, meaning, faith, spiritual leadership, altruism, vision, stakeholder relationships, trust, organizational development, collaboration, and change management. We limited results to peer-reviewed journal articles while excluding commentaries, conference abstracts, and studies focused solely on education and clinical care settings without mention of leadership in the public health space. Scopus was selected because it is the largest multidisciplinary database of peer-reviewed literature and incorporates MEDLINE content, ensuring broad coverage across health sciences and social sciences. EBSCO host was included because it hosts key disciplinary databases such as CINAHL, APA PsycInfo, and Business Source Complete, thereby extending coverage to nursing, psychology, and management literature. Together, Scopus and EBSCO host provide comprehensive coverage of the interdisciplinary domains most relevant to public health leadership. While other databases such as Web of Science and Embase overlap substantially with Scopus, and Global Health (CAB Abstracts) provides more niche international coverage, we determined that Scopus and EBSCO host were sufficient to capture the relevant peer-reviewed studies for this review.

Our review^2^ identified nine studies that focused on Public Health Leadership competencies (Table 3).^3^ To analyze the relevance of these competencies to the practice of Spiritual Leadership, two authors (BW and LF) independently reviewed each study and coded the competencies that aligned with either a necessary or organizational development spiritual leadership competency (Tables 4, 5). A comparative review was then conducted, during which the two authors discussed their independent coding. Discrepancies were resolved through consensus. Competencies for which consensus could not be reached regarding their alignment were excluded from the final mapping.

**Table 3:** Percent of public health competencies related to Spiritual Leadership in the Public Health literature.

Title	Author (Year)	Total number of public health competencies listed	Total number (%) of public health competencies relevant to Spiritual Leadership
Challenges faced by public health nursing leaders in hyper turbulent times	Reyes et al. (81)	4	4 (100%)
Leading the way: competencies of leadership to prevent mis-implementation of public health programs	Moreland-Russell et al. (82)	11	10 (91%)
Not everybody approaches it that way”: nurse-trained health department directors’ leadership strategies and skills in public health	Kett et al. (83)	11	10 (91%)
Leadership in public health: new competencies for the future	Yphantides et al. (84)	9	4 (44%)
Health leadership competency model 3.0	National Center for Healthcare Leadership 3.0 (85)	28	12 (43%)
Leadership for public health 3.0: a preliminary assessment of competencies for local health department leaders	Jadhav et al. (86)	10	4 (40%)
Competencies to guide practice	Strudsholm and Villman (87)	49	19 (39%)
In search for a public health leadership competency framework to support leadership curriculum-a consensus study	Czabanowska et al. (88)	52	20 (38%)
Competency development in public health leadership	Wright et al. (89)	79	22 (29%)

**Table 4:** Public Health leadership literature competencies mapped to Spiritual Leadership competencies.

	**Mindfulness**	**Altruism and Creating a Culture Grounded in Trust**	**Developing Vision**	**Cultivating Stakeholder Relationships**
Czabanowska (88)	Demonstrating the awareness of the impact of your own beliefs, values, and behaviors on your own decision-making and the reactions of others (4)	Encouraging a high level of commitment to the purposes and values of the organization (6)	Creating and communicating a shared vision and inspiring team members to achieve it (2)	Identifying and engaging stakeholders in interdisciplinary projects to improve public health (2)
National Center for Healthcare Leadership (85)	Ability to have an accurate view of one’s own strengths and development needs, including the impact that one has on others (1)	Ability to accurately hear and understand others, especially those who may represent diverse backgrounds and very different worldviews (3)	Ability to lead groups of people toward shared visions and goals (2)	Ability to understand and learn stakeholder formal and informal decision-making structures and power relationships (2)
Jadhav et al. (86)	(0)	Follow ethical standards (1)	(0)	Interacting with interrelated systems (1)
Kett et al. (83)	(0)	Apply core values of inclusivity, empathy, and integrity (1)	Setting the strategic direction and vision (1)	Developing and maintaining partnerships with community organizations (2)
Reyes et al. (81)	Lifelong learning (self-assessment/efficacy & developing) (1)	Valuing diversity, respect, and trust (1)	Articulate a clear vision with strategies to implement (1)	(0)
Moreland-Russell et al. (82)	(0)	Foster an environment of trust (1)	(0)	Sensitivity to communicating with diverse cultures; maintaining excellent relations with our communities (2)
Strudsholm & Vollman (87)	Demonstrating lifelong learning and self-development (3)	Promoting a healthy workplace culture (5)	Garnering support for and momentum to a public health vision (3)	Serving as catalysts to build partnerships and coalitions (3)
Wright et al. (89)	(0)	Modeling effective team leadership traits including integrity, credibility, enthusiasm, commitment, honesty, caring and trust (5)	Encouraging and supporting others to share the vision (2)	Facilitate networking and participation of all stakeholders beyond organizational boundaries (3)
Yphantides et al. (84)	(0)	Putting collective well-being ahead of personal gain (1)	Creating a vision to achieve personal and organizational missions different than the status quo (1)	Awareness of the value of a systems perspective (1)

**Table 5:** Public Health leadership literature competencies mapped to Spiritual Leadership organizational development competencies.

	**Building Teams**	**Managing Change**	**Collaborative Decision Making**	**Managing Conflict**
Czabanowska (88)	Modeling effective group process behaviors including listening, dialoguing, negotiating, rewarding, encouraging, and motivating (3)	Identifying opportunities for change and development of the organization (2)	Sharing views in a non-judgmental, non-threatening way (4)	Effectively use negotiation skills to mediate disputes (2)
National Center for Healthcare Leadership (85)	Ability to work cooperatively and inclusively with other individuals and/or teams they do not formally lead (2)	Ability to energize stakeholders and sustain their commitment to change (2)	(0)	Ability to accurately hear and understand the unspoken or partly expressed thoughts, feelings and concerns, especially those from diverse backgrounds (1)
Jadhav et al. (86)	(0)	Ensure management of organizational change (1)	Collaborate for development (1)	(0)
Kett et al. (83)	Using empathy to communicate and tailor messages and develop a positive reputation in the community (3)	(0)	Inclusive partnerships with the community where the community had power in decision-making (1)	Persistence in the face of adversity (2)
Reyes et al. (81)	Role modeling and mentorship (1)	Collaborative change management (1)	(0)	(0)
Moreland-Russell et al. (82)	Sharing information and responsibility at different organizational levels (2)	Serving as a driving force for change (2)	Offering opportunities for collaborative learning and quality improvement (2)	(0)
Strudsholm & Vollman (87)	Recognizing and encourage contribution of others (3)	Demonstrating understanding of how to guide and be an advocate for change (1)	Possessing effective mediation and negotiation skills (3)	Possessing effective negotiation and mediation skills (1)
Wright et al. (89)	Facilitating empowerment of others to take action (4)	Facilitating, negotiating and collaborating in an increasingly competitive and contentious political environment (3)	Recognize and reconcile emotional and rational elements in collaboration building and strategic planning (4)	Utilizing problem solving, conflict resolution and decision-making skills (2)
Yphantides et al. (84)	(0)	Exerting influence outside of silo to drive change (1)	(0)	(0)

Tables 4, 5 detail how specific Spiritual Leadership competencies map to Public Health Leadership competencies noted in the literature. Each cell includes an example competency from the public health literature alongside the number of total competencies that were relevant in parenthesis. If there were no relevant competencies, we have included (0). For example in Table 4, in the article Czabanowska (87), there were 4 competencies listed related to mindfulness, and we have shown “demonstrating the awareness of the impact of your own beliefs, values and behaviors on your own decision-making and the reaction of others” as the one example competency to include in the table. A full list of the relevant competencies can be found in Appendix 2. The large overlap supports the proposition that Spiritual Leadership competencies are relevant to public health leadership.

Given the relevance of Spiritual Leadership to Public Health Leadership as demonstrated by the overlap in competencies in Tables 3–5, further research can evaluate public health leaders and organizations and track their progress on the competencies for the practice of Public Health Leadership grounded in Spiritual Leadership model.

Advancing empirical research further can investigate how Spiritual Leadership competencies can apply to Public Health Leadership. Fry’s validated Spiritual Leadership Survey (SLS) outlined in Section 3.1 and Appendix 1, which includes scales aligned with these key competencies, has demonstrated strong psychometric properties across diverse organizational contexts, including some health-related settings (23). The SLS offers a reliable means to measure the impact of Spiritual Leadership competencies on outcomes (such as wellbeing, engagement, commitment, and performance). Researchers applying the SLS to assess baseline competency levels in public health leaders and organizations can identify both areas of strength and those needing improvement (e.g., mindfulness habits, trust-building behaviors, or the clarity and resonance of an organization’s vision) to guide targeted leadership development and organizational transformation efforts.

Longitudinal SLS tracking at 12- to 24-month intervals can enable public health organizations to monitor changes in Spiritual Leadership competencies over time to evaluate leadership development interventions and their impact on outcomes (such as reduced burnout, improved collaboration, or enhanced adaptability). Longitudinal studies can establish causal relationships between strengthening Spiritual Leadership competencies and achieving critical public health goals. Research should also explore Spiritual Leadership’s application across a range of public health organizations, including global health systems, government agencies, academic centers, and community-based entities. Comparative studies across these settings can identify which competencies are most impactful in various contexts and how best to tailor interventions. Such research enhances the generalizability of findings and supports the integration of Spiritual Leadership into broader leadership development frameworks.

Importantly, studies can align Spiritual Leadership competencies with public health impact through metrics related to organizational resilience, quality of stakeholder relationships, employee wellbeing and effectiveness in advancing population health outcomes. For example, higher trust and ethical leadership scores may correlate with stronger community partnerships, while greater mindfulness may reduce stress and support ethical decision-making. Linking competency development to such outcomes provides evidence of Spiritual Leadership’s practical relevance to Public Health Leadership. Finally, research should examine how Spiritual Leadership supports values-based organizational change, especially in turbulent environments. Fry and colleagues emphasize that Spiritual Leadership fosters transformation by aligning individual purpose with organizational vision and creating cultures grounded in altruism. In the public health sector—where burnout, complexity, and inequities persist—this alignment may be critical for building sustainable, compassionate, and high-performing systems.

Case studies, pilot studies and eventually meta-analyses, along with mixed methods approaches combining quantitative and qualitative data, can deepen evaluation. In addition to surveys that quantify competency levels, interviews and focus groups can qualitatively explore how Spiritual Leadership competencies are expressed in practice—for example, how reflective practices inform crisis decisions or how a shared vision supports stakeholder alignment. Such designs enhance insight into the mechanisms by which Spiritual Leadership influences organizational culture, resilience, and effectiveness.

In summary, although substantial anecdotal evidence suggests that many public health leaders exhibit Spiritual Leadership values, attitudes, and behaviors, empirical evaluation of Spiritual Leadership competencies using validated tools, mixed-methods, and longitudinal designs can more deeply establish the model’s effectiveness in public health contexts. Such research will clarify how Spiritual Leadership can enhance resilience, collaboration, and purpose—hallmarks of impactful Public Health Leadership.

## 8 Implications for practice

Spiritual Leadership as a moral compass can support a leader’s resilience and growth while fostering inclusive, collaborative approaches that reduce resistance and create sustainable, community-centered efforts; these are especially important for a field with so many challenges and often no clear solutions. To stay resilient and motivated, public health leaders must “start with why” and hold onto their purpose (65).

### 8.1 Mindfulness & reflection

Public health leaders can prioritize mindfulness by incorporating time into their work for reflection for self and community. The time invested supports self-awareness—a needed attribute in public health to make sound decisions. Meditation, journaling or reading affirmations before challenging public meetings, setting aside specific times for self-discovery through conversation and/or personality/leadership assessments are all welcome ways to practice mindfulness. Mentorship and peer coaching can provide critical reflection spaces for leaders to practice transparency within trusted networks before extending those practices more broadly. Through opportunities for individual and shared reflection, leaders can uncover and address hidden assumptions that may hinder open communication and mutual understanding.

### 8.2 Creating vision & shared goals and nurturing stakeholder relationships

In public health workplaces, the leadership team can create a vision document highlighting shared goals that focus on nurturing stakeholder relationships (see section 4.2). As part of this, a formal stakeholder analysis can identify current and future partners, their sources and levels of influence and expectations, and an assessment of the degree to which they are meeting, or exceeding, them. Human resource teams can emphasize to potential employees that they prize colleagues who value compassion, integrity and service, which translates to better stakeholder relationships and improved realization of vision.

### 8.3 Creating a trusting and ethical culture

To create a culture of trust, spiritual leaders in public health can start by modelling the way and openly sharing what gives meaning and purpose in their personal and professional lives. Leaders must continually reinforce ethical norms through their visible behavior, ensuring that compassion, loyalty, and competence are not only encouraged, but also expected, across all relationships. Individual leaders can seek diverse perspectives, admit mistakes, and demonstrate gratitude and patience to reinforce trustworthiness. They can learn and grow from “crucibles of leadership” to deepen their commitment to living a life of meaning and purpose. Then they can purposefully design rituals and structures—such as inclusive dialogue forums, collaborative goal-setting sessions, and recognition programs for altruistic behaviors—that nurture a shared commitment to collective values among all stakeholders. Building and reinforcing the culture allows all employees to believe in, and commit to, hope & faith in a vision of service through a purpose-driven organization.

### 8.4 Organizational development competencies: building teams, managing change, collaborative decision making and managing conflict

Adapting Spiritual Leadership to public health organizations entails working with wide-ranging teams and stakeholders through collaboration, empowerment, and mutual respect, especially as the field regularly involves major decisions that are regularly not the formal responsibility of a single authority (66). Hence, Public Health Leadership necessitates leveraging “soft power” across multiple sectors and different stakeholders.

To manage and promote lasting change, spiritual leaders can engage all public health stakeholders early and often, while including people of all faiths and beliefs from a broad array of voices. Change strategies, such as those of Heifetz, emphasize “encouraging voices from below” (63). Regular reflection, peer feedback, and training in negotiation help leaders balance tensions through uncertainty. By modeling resilience, empowering action, and fostering cultures of adaptability, leaders create environments where change can thrive. Practicing non-judgmental communication, mediation, and negotiation creates “psychological safety” and strengthens the ability to build consensus.

Leaders can apply Spiritual Leadership to navigate conflict by creating shared spaces that encourage open dialogue and respectful resolution of differing views. Spiritual leaders can start by practicing empathetic and active listening and openly summarize the spoken and unspoken concerns being expressed—“what I am hearing in our discussions to date are ….” while regularly asking others to “help me understand” (67). Doing so can signal intent to de-escalate and manage disputes. Persistence during adversity enhances trust and credibility that can help turn conflict into a catalyst for learning, growth, and stronger relationships.

## 9 Conclusion

This paper contributes a review and conceptual framework demonstrating how spiritual leadership can inform and enrich public health leadership theory, education, and practice. Although not an empirical study, it identifies key research gaps and offers directions for future systematic and applied investigations. Public health, often understood as a values-driven profession—a “calling”—can learn from the Spiritual Leadership model which has long been studied and validated for workplace settings. Indeed, Spiritual Leadership could play a foundational role for effective Public Health Leadership by encouraging research and practice pathways in the midst of seemingly insurmountable challenges. Public Health Leadership in an inherently demanding field requires hope & faith, a commitment to something greater than oneself, and a self-awareness of how one’s personal values motivate service to a diverse array of individuals and communities. We encourage the public health community to engage in continued conversations about how Spiritual Leadership can animate and inspire the purpose-driven work to improve the health of all.

## Footnotes

^1^Tables 1, 2 were developed by one of the authors (LF) who is a noted expert in the field of workplace spirituality and spiritual leadership, both as a scholar as well as due to his contribution to Oxford Bibliographies on spiritual leadership (39) as well as his role as associate editor for spirituality and leadership for the *Journal of Management, Spirituality, and Religion*.

^2^While this paper includes a structured search and mapping of published public health leadership competency frameworks, it is not a systematic review or meta-analysis. Instead, it represents a comprehensive conceptual synthesis intended to highlight thematic overlap and inform future empirical inquiry.

^3^Not all the competencies from the studies in Table 3 are considered to map directly into necessary or organizational development spiritual leadership competencies. The Public Health Leadership competencies from these studies not shown as related to Spiritual Leadership in Tables 4, 5 are still relevant for public health leaders to cultivate. However, they are not competences central to the practice of spiritual leadership in public health organizations [e.g., financial skills, talent development, information seeking; see ref. (84)].
