# Supplementary material for: The relevance of Spiritual Leadership to public health: values, meaning and purpose
Source: Front Public Health. 2026 Jan 28;13:1632959. doi: 10.3389/fpubh.2025.1632959 (PMC12892490; doi:10.3389/fpubh.2025.1632959)
Supplement: Supplementary file 1 [file Data_Sheet_1.docx]

**APPENDIX 1**

**Spiritual Leadership Survey Items**

**Please answer the following questions using these responses:**

**1 = Strongly Disagree 2 = Disagree 3 = Neither Agree nor Disagree 4=Agree 5= Strongly Agree**

**Mindful Practice**

- I tend not to notice feelings of tension or discomfort until they really grab my attention.
- I find myself listening to someone with one ear, while thinking about or doing something else at the same time.
- I find it difficult to stay focused on what’s happening in the present.
- I could be experiencing some emotion and not be conscious of it until sometime later.
- I find myself doing things without paying attention.

* These items are reverse scored (5=1, 4=2, 3=3, 2=4, 5=1)

**Spiritual Leadership**

**Vision**

- I understand and am committed to my organization’s vision.
- My organization has a vision statement that brings out the best in me.
- My organization’s vision inspires my best performance.
- My organization’s vision is clear and compelling to me.

**Hope/Faith**

- I have faith in my organization, and I am willing to “do whatever it takes” to ensure that it accomplishes its mission.
- I demonstrate my faith in my organization and its mission by doing everything I can to help us succeed.
- I persevere and exert extra effort to help my organization succeed because I have faith in what it stands for.
- I set challenging goals for my work because I have faith in my organization and want us to succeed.

**Altruistic Love**

- The leaders in my organization “walk the walk” as well as “talk the talk”.
- The leaders in my organization are honest and without false pride.
- My organization is trustworthy and loyal to its employees.
- The leaders in my organization have the courage to stand up for their people.
- My organization is kind and considerate toward its workers, and when they are suffering, want to do something about it.

**Spiritual well-Being**

**Purpose**

- The work I do makes a difference in people’s lives.
- The work I do is meaningful to me.
- The work I do is very important to me.
- My job activities are personally meaningful to me.

**Membership**

- I feel my organization appreciates me, and my work.
- I feel my organization demonstrates respect for me, and my work.
- I feel I am valued as a person in my job.
- I feel highly regarded by my leaders.

**Outcome Variables (Tailored to the Organization’s Needs)**

**Organizational Commitment**

- I feel like “part of the family” in this organization.
- I really feel as if my organization’s problems are my own.
- I would be very happy to spend the rest of my career with this organization.
- I talk up this organization to my friends as a great place to work for.
- I feel a strong sense of belonging to my organization.

**Productivity**

- In my department, everyone gives his/her best efforts.
- In my department, work quality is a high priority for all workers.
- My work group is very productive.
- My work group is very efficient in getting maximum, output from the resources (money, people, equipment, etc.) we have available.

**Satisfaction with Life**

- The conditions of my life are excellent.
- I am satisfied with my life.
- In most ways my life is ideal.
- If I could live my life over, I would change almost nothing.
- So far, I have gotten the important things I want in life.

**Other Economic, Social, and/or Environmental Outcomes of Interest**

**Please identify 1 or more issues that you feel need more attention**

**1.** _______________________________________________________________________

**2.** _______________________________________________________________________

**Other Comments:**

_________________________________________________________________________

**APPENDIX 2**

**Complete and Combined Table 4A and 4B with All Competencies Listed**
